# The Role of Entrapment in Crisis-Focused Psychotherapy Delivered in Psychiatric Emergency Settings: A Comparative Study

**DOI:** 10.3389/fpsyg.2019.02600

**Published:** 2019-11-15

**Authors:** Dana Tzur Bitan, Adi Otmazgin, Mirit Shani Sela, Aviv Segev

**Affiliations:** ^1^Department of Behavioral Sciences, Ariel University, Ariel, Israel; ^2^Shalvata Mental Health Center, Sackler School of Medicine, Tel Aviv University, Tel Aviv, Israel

**Keywords:** crisis intervention, psychotherapy, entrapment, outcome, process

## Abstract

Although many mental health centers offer crisis intervention services as part of their psychiatric emergency facilities, studies assessing outcome, and process of crisis intervention psychotherapy are scarce. One potential psychological construct that might be unique to crisis intervention psychotherapy is entrapment, a psychological construct which reflects an individual’s subjective perception of being in uncontrollable, unremitting, and inescapable circumstances. In this study we aimed to investigate whether changes in entrapment affect the process and outcome of crisis intervention psychotherapy, as compared to its effect in short-term psychotherapy delivered in outpatient units. Sixty-nine patients were recruited for the study. Patients were assessed for level of entrapment, symptoms, well-being, and the working alliance at three time points. The moderating effect of the type of therapy on the associations between changes in entrapment and changes in symptoms, well-being, and the working alliance were assessed using the Hayes process script. The dynamics of change following crisis intervention psychotherapy, as well as the effect of changes in entrapment on symptomatic relief, were illustrated using a clinical vignette of a patient treated in the crisis unit. Results of the moderation analyses indicated that entrapment had a more substantial effect on symptom distress in crisis intervention psychotherapy as compared to its effect in the short-term psychotherapy. Further, the difference in the effect of entrapment across the study groups was manifested primarily in internal entrapment, whereas no moderating effect was found for external entrapment. Clinical vignettes demonstrated the dynamics through which crisis intervention psychotherapy produces changes in entrapment by offering potential outlets from internal thoughts and interpretations of life circumstances. These results suggest that entrapment is a potential underlying process unique to crisis intervention psychotherapy. Limitations, directions for future research, and clinical implications are discussed.

## Introduction

Emergency psychiatric services have recently reported rising numbers of patients in need of immediate care (Owens et al., 2007; Torrey et al., 2012; Lester et al., 2018). This rise in number of referrals to psychiatric emergency services has led many medical centers to develop crisis intervention facilities aimed at delivering emergency psychological and medical care to patients suffering from acute distress. These crisis intervention units offer immediate and proximal interventions delivered by an interdisciplinary team of well-trained professionals in the fields of psychiatry, psychology, social work, and psychiatric nursing (Kowal et al., 2011). Although the settings, treatment approaches, and durations of intervention vary from one unit to another, most of the crisis intervention units also deliver short-term, intensive, and crisis-focused psychotherapy (see Sunderji et al., 2015 for review).

The current formulation of crisis-focused interventions began with the pioneering work of several practicing clinicians who treated survivors of catastrophic situations (e.g., Lindemann, 1944; Rapoport, 1962; Caplan, 1964). These formulations define the goals of crisis intervention to include stabilization, as reflected by the cessation of escalating distress; mitigation of acute signs and symptoms of distress; and restoration of adaptive independent functioning (Flannery and Everly, 2000). Although these are general common goals across most crisis intervention programs, the methods and techniques used to reach these goals tend to develop on an *ad hoc* basis, and are determined pragmatically in response to the patients’ needs. Nonetheless, most of these techniques are not unique to crisis intervention but reflect common aspects of most psychotherapeutic practices, such as establishing a bond and an agreement on therapy goals and aims (Ewing, 1990). Thus, as crisis intervention psychotherapy shares common principles with other psychotherapeutic approaches, it can and should be subjected to scientific research using common psychotherapy research approaches.

Maybe due to the unstructured and varying nature of crisis interventions, the processes and outcomes of crisis-focused psychotherapy delivered in psychiatric emergency settings have not been sufficiently studied. Most studies assessing crisis intervention have focused on the outcomes of these interventions, and in general showed a favorable outcome. For example, Kowal et al. (2011) evaluated the effectiveness of an urgent consultation clinic (UCC) and found significant improvements in life satisfaction, overall functioning, and mental quality of life. Wand et al. (2012) evaluated an emergency-department-based mental health outpatient service and found decreases in patients’ levels of distress. There have also been studies showing that crisis interventions tend to decrease the number of referrals to on-call psychiatrists and overnight inpatient admissions (Parker et al., 2003).

Studies assessing the underlying processes in crisis intervention psychotherapy are scarce. Most of the studies assessing predictors of change have focused on short-term psychotherapy delivered in diverse settings without isolating the crisis nature of some of these interventions. These studies found several potential processes that mediate the effect of psychotherapy on symptom relief, for example changes in defense mechanisms (Kramer et al., 2010), changes in automatic thoughts (Coleman and Casey, 2007), and changes in the working alliance (Gaston et al., 1994). These studies indicate that there are several common processes in crisis intervention psychotherapy which are shared by most psychotherapy approaches, yet no study has previously assessed whether there are specific psychological constructs unique to crisis intervention psychotherapy.

A potential psychological construct which might play an important role in process and outcome of crisis intervention psychotherapy is changes in feelings of entrapment. Entrapment is conceptualized to reflect an individual’s subjective perception of his or her circumstances as being uncontrollable, unremitting, and inescapable (Williams, 2001; Gilbert and Gilbert, 2003). The origins of this construct are grounded within theoretical accounts of high-stress circumstances where escape is motivated but blocked or prevented, and is termed “arrested flight” (Dixon et al., 1989; Dixon, 1998). Entrapment can manifest as either external or internal. External entrapment relates to the perception of events in the outside world as inducing escape motivation. On the other hand, internal entrapment relates to escape motivation that is induced by internal feelings and emotions (Gilbert and Allan, 1998).

Studies assessing the role of entrapment in psychological distress found it to be highly associated with various forms of human psychopathology such as depression, posttraumatic stress disorder (PTSD), and other anxiety syndromes (Taylor et al., 2011; Siddaway et al., 2015). The construct was also previously correlated with predictive factors of depression, such as rumination (Gilbert et al., 2005). Several theoretical frameworks, as well as empirical studies, have suggested that entrapment also serves as a core psychological mechanism in the causal pathways leading from distress to suicidal crises (Li et al., 2018). In a study assessing the prevalence of factors predicting acute suicidal risk among veterans attending urgent care psychiatric clinics, 66% of the sample reported having feelings of entrapment, as identified by their reports of feeling entrapped and with no way out (McClure et al., 2015). These findings suggest that entrapment is associated with high distress circumstances; nonetheless, the effect of entrapment as an important psychological construct during recovery from acute crisis has not been previously examined.

In this study we aimed to assess the predictive value of entrapment on the process and outcome of crisis intervention psychotherapy delivered in psychiatric emergency settings. Our goal was to focus on the measurement of routine processes derived from daily clinical experiences of real-world practice, while employing a practice-based research paradigm (Margison et al., 2000). Specifically, we examined whether the type of treatment (crisis intervention psychotherapy vs. short-term psychotherapy) moderated the association between entrapment and distress and well-being, or affected process measures such as the working alliance. Our main interest was in examining whether there are differential predictors of change among the two interventions provided in the outpatient and the crisis unit. The study hypothesis was that changes in entrapment would be associated with changes in outcome in the crisis intervention psychotherapy, but not in the short-term psychotherapy delivered in the outpatient units. A secondary aim of the study was to explore the differential pattern of changes in external and internal entrapment on symptom distress, well-being, and the alliance.

## Materials and Methods

### Participants

Recruitment to the study was held through two main units of the Shalvata Mental Health Center: the outpatient unit, which treats the majority of outpatients, and the crisis intervention unit, which is an outpatient unit located within the psychiatric emergency department. Patients in the outpatient unit approach the unit via a personal or primary doctor referral. Patients in the crisis intervention unit approach the unit through the referral of either the primary doctor requesting immediate care, or through the referral of the attending psychiatrist in the psychiatric emergency department. Inclusion criteria included the patient being 18 years of age or older, initiation of psychotherapy as determined after an intake or a psychiatric emergency examination, and the patient’s ability and willingness to participate in the study. Exclusion criteria included immediate and proximal suicidal or behavioral risk to the patient or others, or inability to complete self-report assessments.

### Measures

DSM-5 self-rated level 1 cross-cutting symptom measure – adult (American Psychiatric Association, 2013). A self-report measure aimed to assess psychiatric symptoms across 13 domains: depression, anger, anxiety, mania, somatic symptoms, suicidal ideation, psychosis, sleep disturbances, memory, repetitive thoughts and behaviors, dissociation, personality functioning, and substance use. The measure consists of 23 questions about symptoms experienced in the previous 2 weeks. Participants are requested to rate the extent to which they have been bothered by these symptoms on a 5-point Likert scale, ranging from 0 (*not at all*) to 4 (*nearly every day*). The measure was found to have good psychometric properties (Narrow et al., 2013). In order to evaluate total psychopathology severity, total scores were calculated to produce an index of psychopathology. The alpha coefficient in the current sample indicated good internal reliability (Cronbach’s alpha = 0.87 at baseline).

Outcome questionnaire (OQ-45; Lambert et al., 1996). A self-report measure designed to assess patient outcomes during the course of therapy. The scale assesses three primary dimensions: (a) subjective discomfort, (b) interpersonal relationships, and (c) social role performance. The measure is widely used and has been shown to have good internal consistency (0.93), 3-week test–retest reliability (*r* = 0.84), and concurrent validity (Snell et al., 2001). The OQ-45 was previously found to be sensitive to changes in both clinical outpatient populations, and in severely ill inpatient samples (Lambert et al., 1996; Doerfler et al., 2002). For the purposes of the current study, we used the OQ-45 as a measure assessing outcome at three time points: before, mid-point, and after therapy. The alpha coefficient in the current sample indicated high internal reliability (Cronbach’s alpha = 0.91 at baseline).

Entrapment Scale (Gilbert and Allan, 1998). The Entrapment Scale (Gilbert and Allan, 1998) is a 16-item self-report instrument assessing perceptions of psychological entrapment. The scale includes two subscales, with 6 items tapping internal entrapment (perceptions of entrapment by one’s own thoughts and feelings) and 10 items related to external entrapment (perceptions of entrapment by external situations). Ratings are made on a 5-point scale, scored from 0 to 5, with higher scores indicating greater entrapment. The Entrapment Scale was found to have good psychometric properties (Gilbert and Allan, 1998; Taylor et al., 2011). The alpha coefficient in the current sample indicated excellent internal reliability (Cronbach’s alpha = 0.94 at baseline).

The 6-item Session Alliance Inventory (SAI, Falkenström et al., 2015). The SAI is a self-report measure of working alliance, aimed at assessing agreement between patients and therapists on treatment goals and therapeutic tasks, and at assessing the level of positive emotional bond. The SAI is highly correlated with the full version of the working alliance inventory (Horvath and Greenberg, 1994) and the working alliance inventory-short revised (WAI-SR, Hatcher and Gillaspy, 2006), and has a high reliability for the composite sum or mean of the six items (Falkenström et al., 2015). The original version of the working alliance inventory was previously found suitable for detecting weekly changes in alliance, and to be associated with changes in therapy outcome (Klein et al., 2003). The short version of the SAI was also previously utilized for the detection of changes in alliance in naturalistic psychiatric settings and was found to adequately detect such changes (Tzur Bitan et al., 2019). The alpha coefficient in the current sample indicated good internal reliability (Cronbach’s alpha = 0.89 at baseline).

Schwartz Outcome Scale (SOS-10; Blais et al., 1999). The SOS-10 is a 10-item scale designed to measure psychological health and well-being. Items are rated on a 7-point Likert scale with higher total scores indicating better psychological well-being. The measure has been used in a range of settings (Young et al., 2003) and has demonstrated high internal consistency (Beard and Björgvinsson, 2014). Internal consistency in the current sample was adequate (Cronbach’s alpha = 0.83 at baseline).

### Procedure

The study was authorized by the Shalvata Mental Health Center Institutional Review Board (IRB), approval number 0017-17-SHA. Patients in the outpatient and the crisis intervention units were recruited by the study coordinator. In the outpatient units, patients were allocated to receive psychotherapy by the treating staff after an intake meeting. After the intake they were approached by the study coordinator, who provided information about the study. Patients from the crisis intervention unit were recruited after the initial psychiatric emergency examination. Assignment to either the crisis or the outpatient unit was based on level of urgency. Patients in need of an immediate intervention were assigned to the crisis intervention, while patients in need of non-urgent intervention were referred to the outpatient unit. The establishment of the crisis unit was conceptualized to be practice-research oriented; therefore, assessments were delivered for clinical purposes as part of the clinical routine. Patients were informed regarding the clinical and empirical utility of the assessments and were given the choice to participate. Informed consent was waived due to the clinical usage of the assessments. Initiation of the comparative study was conducted a few months after the establishment of the crisis intervention unit. After receiving IRB approval, patients from the outpatient unit were recruited and signed informed consent. Short-term psychotherapy included integrative psychotherapy combining dynamic, cognitive, and behavioral interventions. In the crisis intervention psychotherapy, the therapists delivered crisis-focused therapy aimed to specifically address the presenting and current crisis as outlined in the Flannery and Everly (2000) review. The psychotherapeutic approach during sessions, aimed to achieve the alleviation of a psychological crisis, as specified in the Flannery and Everly (2000) review, was primarily integrative, and included elements of contemporary psychodynamic psychotherapy (e.g., Summers and Barber, 2009), as well as cognitive and behavioral elements. In both the crisis-focused psychotherapy and the short-term psychotherapy the number of sessions was up to 16, with a maximum overall duration of psychotherapy lasting 3 months. Sessions were held once a week, but in the crisis intervention psychotherapy, the frequency of sessions could have been higher as a function of the patient’s needs. Therapy sessions began shortly after the intake meeting. Assessments were performed after the first psychotherapy session (baseline T1), a month and a half after the initiation of treatment (mid-therapy, T2), and at the end of the therapy (T3). Each patient was followed for a period of 3 months. The primary outcome measure was the DSM-5 self-rated level 1 cross-cutting symptom measure, while the OQ-45, and the SOS-10 were secondary outcome measures. Process measures included the entrapment scale and the SAI. Overall, the duration of patient recruitment lasted a total of 2 years (2016–2018).

### Statistical Analyses

Demographic and clinical baseline differences between the outpatient and crisis intervention patients were assessed using chi square tests for categorical variables and *t*-tests for continuous variables. In order to assess the moderating effect of the type of psychotherapy (short-term psychotherapy vs. crisis intervention psychotherapy) on the association between changes in entrapment and changes in outcome, we first calculated the differences in all outcome variables from baseline to T3. For the purposes of the moderation analysis, we considered changes from pre- to post-treatment (baseline to T3) on all outcome and process measures. Mid-treatment scores were utilized for empirically assessing trajectories of change for the case report, as well as for individualized grown curves. Tests of normality were performed on all outcome and processes variables using the Shapiro–Wilk test of normality. The Hayes (2012) process script was then used to assess the moderating effect of the group (short-term vs. crisis intervention) on the predictive effect of entrapment on the various outcome measures. Bootstrap resamples were utilized to test the moderation effects in cases where normality assumptions were violated (Preacher and Hayes, 2008). All statistical procedures were conducted using SPSS version 25.

## Results

### Participants

**FIGURE 1:**
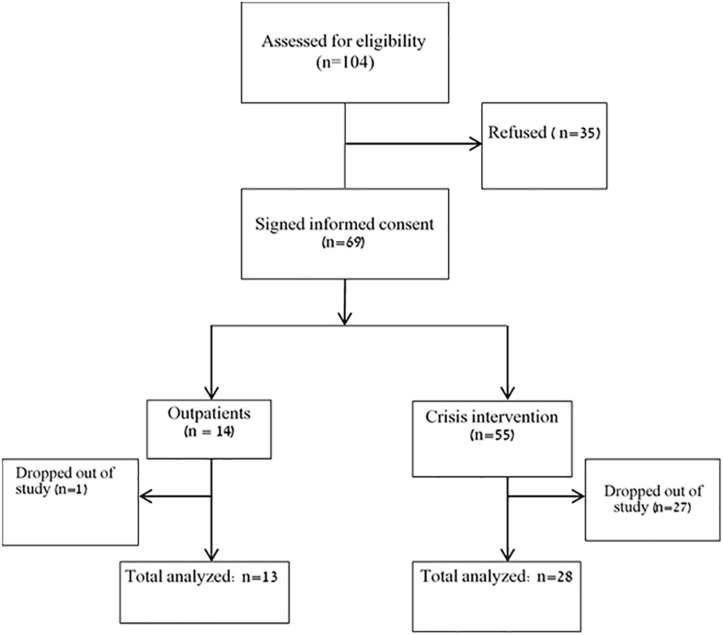
Recruitment process of study participants.

A flowchart of patients’ recruitment process is presented in Figure 1. Overall, 104 patients were assessed for eligibility, out of whom 35 refused to participate. Of the total 69 patients who participated in the study, 15 patients completed all three assessments in the crisis intervention unit, and an additional 13 completed two assessments. In the outpatient units, 12 completed all three assessments, and one completed two assessments. For the purpose of the current study, we analyzed patients with at least two complete measurements, resulting in a total sample of 41 patients.

Clinical and demographic characteristics of participants in the outpatient and crisis intervention units are presented in Table 1. The mean age was 40.1 (*SD* = 14.7). Of the total number, 51.2% were men and 48.8% were women. No significant differences in age or gender were found across the study groups. The main diagnostic clusters of study participants included adjustment disorders (26.8%), anxiety disorders (26.8%), PTSDs (19.5%), depression (22.0%), and other diagnoses such as eating disorders and schizophrenia (4.9%). There were significant differences in diagnosis distribution across the two groups, reflected by a higher prevalence of adjustment disorders in the crisis intervention group as opposed to a higher prevalence of anxiety disorders in the outpatient group, χ ^2^ (4) = 13.58, *p* ≤ 0.001. Comorbidity was diagnosed in 19.5% of the study participants; most of the patients received psychiatric medications during treatment (82.9%), with no significant differences between groups in prevalence of comorbidity or medication utilization. The average time in research was 83.80 days (*SD* = 49.1), with the outpatient group showing a significantly longer time in research compared to the crisis intervention group, *t*(39) = −3.86, *p* < 0.001).

**TABLE 1:** Demographic and clinical characteristics of study participants.

	**Crisis intervention psychotherapy (*n* = 28)**	**Short-term psychotherapy (*n* = 13)**	***p***
Age (mean, SD)	42.64 (14.78)	34.62 (13.46)	0.1
Gender			0.37
Male	13 (46.4%)	(61.5%) 8	
Female	(53.6%) 15	(38.5%) 5	
Total time in research (days)	(35.57) 66.49	121.31 (54.83)	*p* < 0.001
Comorbidity^*a*^	(21.4%) 6	2 (15.4%)	0.65
Concurrent medication	(89.3%) 25	9 (69.2%)	0.11
Main diagnosis			*p* < 0.001
Adjustment	10 (35.7%)	1 (7.7)	
Anxiety	(10.7%) 3	8 (61.5%)	
PTSD	(21.4%) 6	2 (15.4%)	
Depression	8 (28.6%)	1 (7.7%)	
Others	1 (3.6%)	1 (7.7%)	

*^*a*^Comorbid diagnosis included personality disorders, attention deficit and hyperactivity disorders, adjustment disorders, PTSD, and agoraphobia.*

### Baseline Clinical Characteristics of the Study Groups

No significant baseline differences were observed among the two study groups in general symptomatology, symptom distress, interpersonal and social relations, internal and external entrapment level, or working alliance (see Table 2). A significant difference, *t*(38) = −3.29, *p* ≤ 0.01, was found in the quality of life measure (SOS-10), indicating that the outpatient group reported higher quality of life at baseline (*M* = 33.32, *SD* = 10.67) compared to that reported by the crisis intervention group (*M* = 21.96, *SD* = 9.89). With regards to the main outcome measure (the DSM symptom measure), one patient from the crisis unit did not complete the DSM-5 measure at baseline, additional 13 patients from the crisis unit did not complete the measure at post-treatment, and one patient from the outpatient unit did not complete the measure at post-treatment. Incomplete items were excluded from analysis. Overall, patients in both units exhibited symptomatic improvements from baseline to post-treatment, as manifested in a significant change in DSM symptoms of interpersonal relations [short-term, *t*(12) = 1.80, *p* < 0.05; crisis intervention, *t*(26) = 2.74, *p* < 0.001] and hopelessness [short-term, *t*(12) = 1.81, *p* < 0.05; crisis intervention, *t*(27) = 1.54, *p* < 0.05] from pre-treatment to mid-treatment; and improvement in symptoms of worry and preoccupation [short-term, *t*(11) = 1.91, *p* < 0.05; crisis intervention, *t*(14) = 3.30, *p* < 0.001]; and social relations [short-term, *t*(11) = 1.77, *p* < 0.05; crisis intervention, *t*(14) = 3.37, *p* < 0.001] from mid-treatment to post-treatment.

**TABLE 2:** Baseline clinical characteristics of the study groups (*n* = 41).

**Measure**	**Crisis intervention (*n* = 28)**	**Outpatient (*n* = 13)**	**Bootstrap 95% CI**	***P***
Adult DSM-V symptom	35.73 (14.16)	27.23 (17.27)	−18.80; 1.80	0.10
OQ-45 general score	76.94 (28.08)	66.15 (31.59)	−30.93; 9.35	0.29
OQ-45 symptom distress	48.71 (18.79)	39.92 (22.82)	−22.67; 5.10	0.20
OQ-45 interpersonal relations	15.35 (8.58)	15.92 (7.7)	−5.13; 6.29	0.84
OQ-45 social relations	12.88 (6.75)	10.3 (5.36)	−6.93; 1.78	0.24
Entrapment Scale	34.04 (19.8)	24.62 (21.89)	−23.31; 4.47	0.18
Internal entrapment	20.86 (11.86)	14.92 (12.11)	−9.92; 2.95	0.15
External entrapment	13.18 (9.08)	9.69 (10.36)	−14.03; 2.17	0.28
SAI	35.29 (5.81)	30.54 (8.39)	−9.70; 0.20	0.06
SOS-10	21.96 (9.89)	33.23 (10.67)	3.97; 18.56	*p* < 0.01

### Moderation Analysis

The Shapiro–Wilk test of normality indicated a normal distribution of all process and outcome variables (Shapiro–Wilk statistic ranging from 0.95 to 0.98, non-significant), with the exception of the interpersonal subscale of the OQ-45 which produced a statistic of 0.88, *p* < 0.01. Results of the simple effects of all moderation analyses are presented in Table 3.

**TABLE 3:** Moderation analyses for the effect of type of psychotherapy on the association between entrapment and therapy outcomes.

		**Effect**	**SE**	***T***	**95% CI**	***P***
Adult DSM-V symptoms	Short-term	0.32	0.18	1.78	−0.04; 0.69	0.083
	Crisis intervention	0.62	0.09	7.19	0.44; 0.79	<0.001
OQ45-sum	Short-term	0.62	0.31	1.98	−0.01; 1.26	0.054
	Crisis intervention	1.30	0.15	8.73	1.00; 1.61	<0.001
OQ symptom distress	Short-term	0.45	0.18	2.42	0.07; 0.84	0.02
	Crisis intervention	0.96	0.09	10.61	0.78; 1.14	<0.001
OQ social relations	Short-term	0.08	0.12	0.66	−0.16; 0.32	0.50
	Crisis intervention	0.15	0.05	2.56	0.03; 0.26	<0.05
OQ interpersonal relations	Short-term	0.08	0.11	0.77	−0.13; 0.31	0.44
	Crisis intervention	0.19	0.05	3.73	0.09; 0.3	<0.001
SOS-10	Short-term	–0.16	0.20	–0.76	−0.58; 0.26	0.44
	Crisis intervention	–0.54	0.10	–5.37	−0.74; −0.33	<0.001
SAI	Short-term	0.16	0.15	1.06	−0.14; 0.47	0.29
	Crisis intervention	–0.09	0.07	–1.24	−0.24; 0.05	0.22

**FIGURE 2:**
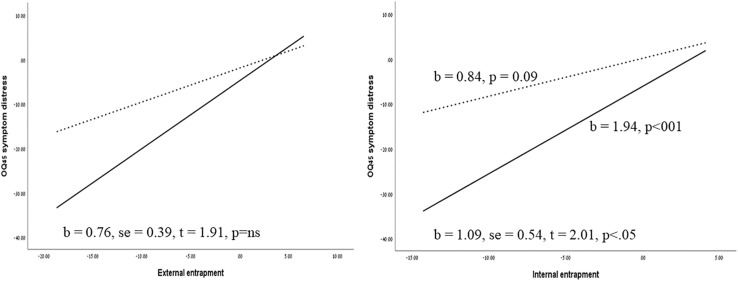
Simple regression slopes of the prediction of symptom distress by internal and external entrapment at the crisis intervention unit versus the outpatient group. Solid lines represent the crisis intervention psychotherapy group and dashed lines represent the outpatient group. The regression slopes refer to the prediction of changes from pre-treatment to post-treatment (Tl–T3).

As can be seen in Table 3, for the OQ-45 total score, a marginally significant moderating effect of type of therapy was found (*B* = 0.68, *t* = 1.96, 95% CI: −0.02;1.39, *p* = 0.057), where changes in entrapment were more predictive of changes in total distress scores in the crisis intervention psychotherapy group (*B* = 1.30, *p* < 0.001) as compared to the short-term psychotherapy outpatient group (*B* = 0.62, *p* = 0.054). Moderation analyses conducted on the OQ45 subscale indicated a significant moderating effect of type of therapy on symptom distress subscale (*B* = 0.51,SE = 0.20, *t* = 2.41, *p* < 0.05, 95% CI 0.07–0.93), indicating a significant effect of changes in entrapment on symptom distress in the crisis intervention group (*B* = 0.96, *p* < 0.001), and a lower significant effect in the outpatient group (*B* = 0.45, *p* < 0.05). No significant moderation effect was found for the other subscales of the OQ45, or for the DSM-V symptom scale, the SAI, or the SOS-10 quality of life measure. As the changes in the OQ-45 interpersonal relations subscale were not normally distributed, bootstrap confidence intervals were calculated with 5,000 re-samplings to test the moderating effect. The results indicated a non-significant interaction effect (effect = 0.11, Boot SE = 0.13, Boot 95% CI −0.08; −0.43). In order to probe the effect of type of therapy on the association between entrapment and symptom distress, we tested both internal and external entrapment on this outcome variable. The analyses indicated a significant moderation effect of type of therapy on internal entrapment and symptom distress (*B* = 1.09, se = 0.54, *t* = 2.01, *p* < 0.05, 95% CI −0.01; −2.2), according to which a significant effect of internal entrapment on symptom distress was found in the crisis intervention psychotherapy group (*B* = 1.94, *p* < 001), yet no significant effect of internal entrapment was found in the outpatient group (*B* = 0.84, *p* = 0.09). No moderating effect was found for external entrapment. Figure 2 displays the simple regression slopes of the prediction of symptom distress by internal and external entrapment in the crisis intervention unit versus the short-term psychotherapy group.

### The Dynamics of Symptomatic Change Following Changes in Entrapment in Crisis Intervention Psychotherapy: A Case Illustration

To provide an in-depth exploration of the dynamics of change during crisis intervention psychotherapy, as well as the clinical manifestations of changes in entrapment and their effect on symptomatic relief, we describe here a clinical course of crisis intervention psychotherapy delivered in the setting of the psychiatric emergency unit. The description demonstrates the unique features of crisis intervention psychotherapy as a tool to alleviate entrapment, and possible trajectories leading from changes in entrapment to relief of distress. All personal identifiers in the following clinical demonstration have been removed or extensively disguised, so that the patient is not identifiable and cannot be identified through the clinical description.

Victoria is a 45-year-old woman, recently divorced and with two children. In the past, she had worked as a litigator in a large litigation firm where she had interned during law school. Victoria lost her father when she was a very young child; her mother never remarried, and she mourned the death of her husband throughout most of her adult years. Victoria has one older sister, whom she described as passive and submissive. Her mother raised both of them, as Victoria described, due to her sense of obligation, but was mostly absorbed in her grief, and Victoria felt that her mother had no real interest in her. Thus, Victoria spent most of her childhood feeling unwanted, “like something you have no choice but to handle,” and alone. Throughout her mature life, she constantly felt that she needed to resolve her problems on her own, and that no one had any real interest in her. When Victoria met her husband, she felt for the first time that she could trust someone else to care for and about her. This relationship enabled her to trust others for the first time, and gradually she became more socially active and made new friends. The sense of belonging and the security she felt with her husband allowed her to pursue her dream to become a lawyer, and she completed her degree successfully. Victoria and her husband had been married for 20 years, but in the last few years had struggled with issues of trust and loyalty. During these years, Victoria started to wonder whether people were truthful, and she began to question the motives of her colleagues and friends. Although she was exposed to interpersonal conspiracies and manipulative behavior at work, she tended to split her personal and professional lives and could not stand the idea that her closest friends might be manipulative or dishonest toward her. Gradually she started to distance herself from others and only confided in her closest family members. This gradual alienation from others resulted in feelings of loneliness, dissatisfaction with her work, reduced social activities, and negative mood. Victoria went to see several psychotherapists and psychiatrists, but felt that their treatment approach was either wrong or not suited to her needs. She arrived at the psychiatric ER with one of her friends, after confiding her suicidal thoughts in her, requesting that she keep her children safe. During the psychiatric examination, Victoria refused to be admitted to a psychiatric ward, but agreed to receive psychotherapy in the crisis intervention unit.

During the first session with the therapist, Victoria expressed a sense of hopelessness, a feeling that she couldn’t trust others, and questioned whether the treatment could be of help. She repeatedly said that she didn’t understand how talking about her problems would help her resolve them. Nonetheless, she could not think of any other alternative to help her handle her problem and stated that “coming to the ER was the only thing I could think of.” Noticing the distrustful attitude, the therapist focused on the establishment of an alliance with Victoria, while stating that as dealing with the problem by herself had not produced the desired outcome, she might as well give the therapy a chance. The sessions were initially focused on the validation of her frustration and despair, but at the same time on the idea that coming to the ER was an active step that she had made to resolve her problems. In the therapeutic relations, the therapist frequently conceptualized the therapeutic sessions as a mutual and collaborative search for potential outlets for her difficulties. Therapy sessions gradually shifted to a discussion of her loneliness, as well as the contradictory feelings of wanting to be with others while simultaneously being unable to trust them. The following clinical vignette demonstrates the use of therapy as a means of resolution of feelings of loneliness, as well as the collaborative efforts to find outlets for her negative emotions:

Victoria: What I am now is very different from what I used to be. I can’t recognize myself anymore.
Therapist: How so?
Victoria: I used to be a very friendly person; now I can’t even engage in a discussion with my own family. I feel so distant from the conversation.
Therapist: Why is that?
Victoria: I think I’m too absorbed in my own problems. I keep on thinking: What will be the end of this mess, when will I get better? These are issues I can’t discuss with my children or with my family.
Therapist: So you say that you have no one to confide in.
*Victoria: Yes. Well*… *I have you.*
(*pause*)
Therapist: Yes, you do have me (pause). I think it’s an important thing you are now noticing. And I would also like to remind you that your friends were worried about you, and they brought you here. So in a sense, the fact that you confided in them in a time of need enabled you to reach out for help and be supported.
Victoria: I did not ask them to do that.
Therapist: Well maybe you did not view them as a source of support.
Victoria: I used to tell my friends everything, but it became so complicated when I got divorced. But you are right that they are still my friends. They still call me every day, you know.
Therapist: Well, maybe if you try to reconnect with them then you will have more sources of support, and then maybe you can also find time and energy to interact with your family and friends as you did before.
Victoria: I guess I can try that.

**FIGURE 3:**
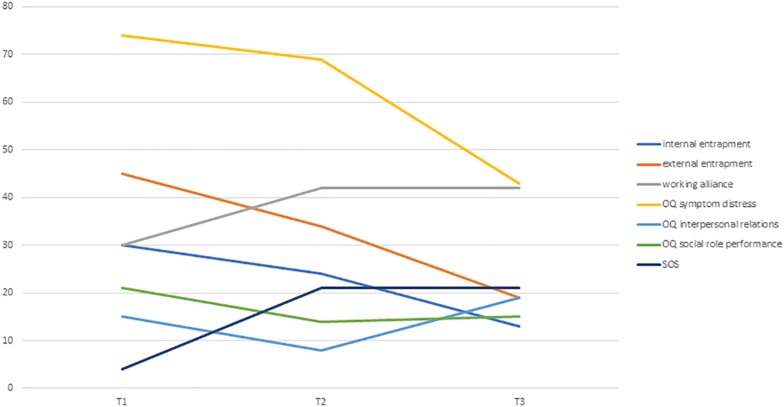
Changes in internal and external entrapment, alliance, and symptoms in Victoria’s reports.

**FIGURE 4:**
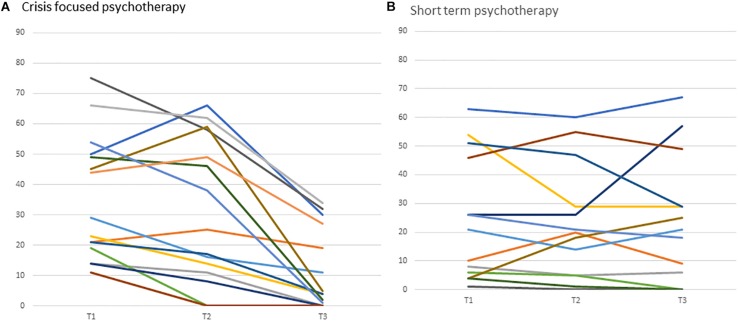
Individual grown curves of changes in entrapment in crisis intervention psychotherapy versus usual care. Curves in panel **(A,B)** represent individual patients and their trajectory of change in entrapment from baseline (Tl) to post-treatment (T3).

The clinical vignette demonstrates the formulation of the therapeutic bond as a potential outlet for Victoria’s feelings of having no support, as well as demonstrating the way that the collaborative endeavor enables a reconnection to feelings of vitality and importance. Figure 3 illustrates the changes in internal and external entrapment, alliance, and symptoms in Victoria’s reports. Figure 4 presents the individualized growth curves of changes in entrapment across participants in the crisis intervention psychotherapy versus treatment as usual. As can be seen, in the crisis intervention psychotherapy the majority of the patients showed a clear reduction in entrapment, while the majority of the patients in the short-term psychotherapy did not show the same reductions.

## Discussion

In this study we aimed to assess the effect of entrapment on the outcomes of crisis intervention psychotherapy delivered in psychiatric emergency settings, as compared to its effect in short-term psychotherapy delivered in the outpatient unit. The results of the moderation analyses indicated that entrapment had a more substantial effect on symptom distress in the crisis intervention psychotherapy group as compared to its effect in the short-term psychotherapy outpatient group. Further, we found that the difference in the effect of entrapment across the study groups was manifested primarily in internal entrapment, whereas no moderating effect was observed for external entrapment.

Although type of therapy was only significant in moderating the effect of entrapment on symptom distress, a review of the simple slope effects indicates that entrapment had a more substantial effect on most outcome variables in the crisis intervention psychotherapy group, whereas its effect in the outpatient unit was either smaller or non-significant. These results suggest that this psychological construct, which has been associated primarily with suicidal crises, also shares common characteristics with situations of extreme distress, where a need for immediate psychological intervention arises. Theoretical models focusing on the effect of entrapment on suicidal behavior might indicate several possible shared mechanisms. For example, the “cry of pain” model suggested by Williams (2001) postulates that suicidal behavior is a response to the presence of defeat, the perception of no escape, and the perception of no rescue. Thus, the predictive effect of changes in entrapment on therapy outcomes in crisis intervention psychotherapy might represent the perception of the therapeutic intervention as a possible means of rescue or escape from unbearable emotions. As such, it is possible that the formulation of crisis intervention, as well as the intensive attunement to the distressful event associated with the crisis, lead to a more pronounced effect of entrapment during crisis intervention, as opposed to its role in short-term psychotherapy. The case of Victoria further illustrates how a therapeutic bond in times of crisis can serve as a temporal means for escaping feelings of loneliness, as well as a secure base from which to explore different alternatives toward the resolution of origins of distress.

The association between changes in entrapment and changes in symptom distress further strengthen the conceptual importance of entrapment, as it suggests that entrapment is not only predictive of acute crises, but also has an important contribution in facilitating recovery from them. Although the effect of changes in entrapment during psychological intervention has not been previously studied, several authors have noted the need to target entrapment as a possible mediating component of psychotherapy outcomes (O’Connor et al., 2013; Li et al., 2018). According to these suggestions, entrapment can serve as a psychological mechanism which mediates the transition from suicidal ideation to suicidal attempt when accompanied by intense negative affect, loss of cognitive control, hyper-arousal, and social withdrawal. The results of the current study indicate that to a certain extent, the formulation of a crisis intervention enables the modification of entrapment, thereby reducing the intensity of negative emotions. Thus, this intervention can be viewed as a possible preventative intervention, aimed at limiting the possible negative effects of extreme distress by offering alternative paths of action. This possible trajectory should be subjected to additional research.

An additional finding that emerged from our study is that the association between internal entrapment and outcome was moderated by the type of therapy, whereas external entrapment was not. These findings indicate that the unique contribution of crisis intervention, and specifically its unique role in entrapment, is manifested by its effect on internal entrapment. The concept of internal entrapment – which conveys the feeling of being trapped within one’s own thoughts and feelings – has been primarily associated with defeat (Owen et al., 2018) and rumination (Gilbert et al., 2005). These associations suggest that the essence of a crisis dominated by internal entrapment is embodied in patients’ thoughts and interpretations of their life circumstances, and that entrapment occurs when the patient conceives no way out of these thoughts. The results of our study further extend these findings, as they suggest that psychotherapies targeting the current crisis might produce relief primarily by targeting this ruminative state, and not necessarily by intervening with external life circumstances.

This study has several important theoretical and clinical implications. To the best of our knowledge, this is the first study that has addressed entrapment not only as a construct predictive of psychopathology, but also as a construct predictive of psychotherapy outcome. The findings of the current study indicate that changes in entrapment are associated with changes in outcome, and that this association is evident in crisis intervention psychotherapy but not in short-term psychotherapy. These findings suggest that entrapment is an important construct in crisis-focused psychotherapy and may even constitute a mechanism of change in this specific intervention. Future studies should assess the mediating role of entrapment in crisis-focused psychotherapy in order to assess its potential role as a mechanism of change, while utilizing a study design which enables the determination of causality (Kazdin, 2007). For example, it can be hypothesized that the collaborative act of formulating and disentangling the essence of the crisis allows the patient to switch from a static mental position to a dynamic one, which then allows for the resolution of the crisis and movement toward more constructive solutions. Additional studies are needed to assess potential mediating effects of specific interventions delivered in crisis intervention psychotherapy and their effect on entrapment, as well as the trajectories by which they affect the outcome. For example, it can be hypothesized that when crisis intervention psychotherapy is initiated, patients’ levels of hope and confidence in their ability to recover from their distress are elevated. It can also be hypothesized that the working alliance mediates the influence of entrapment on therapy outcome (Dunster-Page et al., 2018), such that the mutual bond targeting the resolution of the crisis leads to symptom reduction. For example, it can be speculated that therapists working in the crisis intervention mode were able to deal with entrapment via the construction of a good therapeutic alliance despite the entrapment of the patients. Additional studies employing a larger sample size should assess such a potential mediating effect. The current study indicates that entrapment may serve as a psychological component that is relevant not only in cases of suicidal ideation, but also in situations of acute distress. Although entrapment has been previously found to be associated with several psychopathologies (Siddaway et al., 2015), no study has demonstrated its effect in patients presenting with acute distress. Clinically, the results of our study suggest that crisis intervention models might benefit from explicitly relating to the concept of entrapment, and from incorporating ongoing evaluations of this construct during psychological interventions. Such interventions can include an active assessment of feelings of entrapment as well as the construction of settings for the intervention to include the mutual search for alternative solutions.

This study has several limitations. First, due to the naturalistic nature of the study, as well as the nature of crisis events which frequently affect the ability to recruit patients, the sample size is considered small. Future studies should assess the effect of entrapment on crisis psychotherapy and regular care with a larger sample size. As there were significant differences in diagnoses across the study groups, with the crisis intervention group showing more adjustment disorders and the outpatient units showing more anxiety disorders, these differences may account for the differences in the effect of entrapment across the study groups. Although this confounder cannot be excluded, the differences between anxiety and adjustment disorder are not extreme, as they are often associated, and individuals with one or the other present similar clinical symptoms (Carta et al., 2009); therefore, the differences in diagnoses are not likely to account for our findings. Baseline differences between the study groups were also found in quality of life; thus, it is possible that these differences masked the ability to detect differential changes in this variable. Additional studies are needed in order to further assess the effect of crisis intervention on quality of life, compared to short-term psychotherapy. Finally, it is often argued that the concept of “crisis” lacks a specific operational definition. Thus, it can be postulated that the mere referral to psychiatric emergency does not necessarily constitute a crisis. Although this problem with the operational definition of crisis is common to most crisis intervention studies, future research should attempt to clarify the validity of this definition, as well as to assess the level of generalizability of our findings to other populations suffering from severe distress. Taken together, our study sheds light on entrapment as an important psychological factor involved in facilitating therapeutic change, and should therefore be incorporated in process psychotherapy research as well as in clinical practice.

## Data Availability Statement

The datasets generated for this study will be made available upon request to the corresponding author.

## Ethics Statement

The studies involving human participants were reviewed and approved by the Institutional Review Board of the Shalvata Mental Health Center. The patients/participants provided their verbal and written informed consent to participate in this study. Written informed consent was obtained from the individual(s) for the publication of any potentially identifiable images or data included in this article.

## Author Contributions

DT and AS initiated and designed the study, executed and acquired the data, and critically revised the manuscript. AO executed and acquired the data and drafted the manuscript. MS acquired the data and critically revised the manuscript.

## Conflict of Interest

The authors declare that the research was conducted in the absence of any commercial or financial relationships that could be construed as a potential conflict of interest.
